# Transcriptome analysis of skeletal muscle in dermatomyositis, polymyositis, and dysferlinopathy, using a bioinformatics approach

**DOI:** 10.3389/fneur.2023.1328547

**Published:** 2023-12-06

**Authors:** Ha-Neul Jeong, Taek Gyu Lee, Hyung Jun Park, Young Yang, Seung-Hun Oh, Seong-Woong Kang, Young-Chul Choi

**Affiliations:** ^1^Department of Neurology, Yonsei University College of Medicine, Seoul, Republic of Korea; ^2^Brain Korea 21 PLUS Project for Medical Science, Yonsei University, Seoul, Republic of Korea; ^3^Research Institute of Women's Disease, Sookmyumg Women's University, Seoul, Republic of Korea; ^4^Department of Neurology, CHA Bundang Medical Center, School of Medicine, CHA University, Seongnam-si, Republic of Korea; ^5^Department of Rehabilitation Medicine, Gangnam Severance Hospital, Yonsei University College of Medicine, Seoul, Republic of Korea; ^6^Rehabilitation Institute of Neuromuscular Disease, Yonsei University College of Medicine, Seoul, Republic of Korea

**Keywords:** dermatomyositis, polymyositis, dysferlinopathy, transcriptome, bioinformatics

## Abstract

**Background:**

Polymyositis (PM) and dermatomyositis (DM) are two distinct subgroups of idiopathic inflammatory myopathies. Dysferlinopathy, caused by a dysferlin gene mutation, usually presents in late adolescence with muscle weakness, degenerative muscle changes are often accompanied by inflammatory infiltrates, often resulting in a misdiagnosis as polymyositis.

**Objective:**

To identify differential biological pathways and hub genes related to polymyositis, dermatomyositis and dysferlinopathy using bioinformatics analysis for understanding the pathomechanisms and providing guidance for therapy development.

**Methods:**

We analyzed intramuscular ribonucleic acid (RNA) sequencing data from seven dermatomyositis, eight polymyositis, eight dysferlinopathy and five control subjects. Differentially expressed genes (DEGs) were identified by using DESeq2. Enrichment analyses were performed to understand the functions and enriched pathways of DEGs. A protein–protein interaction (PPI) network was constructed, and clarified the gene cluster using the molecular complex detection tool (MCODE) analysis to identify hub genes.

**Results:**

A total of 1,048, 179 and 3,807 DEGs were detected in DM, PM and dysferlinopathy, respectively. Enrichment analyses revealed that upregulated DEGs were involved in type 1 interferon (IFN1) signaling pathway in DM, antigen processing and presentation of peptide antigen in PM, and cellular response to stimuli in dysferlinopathy. The PPI network and MCODE cluster identified 23 genes related to type 1 interferon signaling pathway in DM, 4 genes (*PDIA3, HLA-C, B2M, and TAP1*) related to MHC class 1 formation and quality control in PM, and 7 genes (*HSPA9, RPTOR, MTOR, LAMTOR1, LAMTOR5, ATP6V0D1, and ATP6V0B*) related to cellular response to stress in dysferliniopathy.

**Conclusion:**

Overexpression of genes related to the IFN1 signaling pathway and major histocompatibility complex (MHC) class I formation was identified in DM and PM, respectively. In dysferlinopathy, overexpression of *HSPA9* and the mTORC1 signaling pathway genes was detected.

## 1 Introduction

Idiopathic inflammatory myopathies (IIMs) comprise a heterogeneous group of autoimmune conditions characterized by skeletal muscle weakness and inflammation. The main clinical subtypes of IIM are classified as follows: dermatomyositis (DM); polymyositis (PM); immune-mediated necrotizing myopathy (IMNM); overlap syndrome with myositis (overlap myositis [OM]), including anti-synthetase syndrome (ASS); and inclusion body myositis (IBM) (1). The pathogenic mechanisms underlying the different myositis types and subtypes remain unclear. In recent years, different approaches, including genomic, epigenetic, transcriptomic, proteomic, and autoantibody studies, have been used to understand IIM pathogenesis (2).

Studies involving expression profiling of patients with DM or PM showed that the genes related to major histocompatibility complex (MHC) class I (MHC-I), MHC class II (MHC-II), cytokines, adhesion molecules, the actin cytoskeleton, and immunoglobulins were differentially expressed in muscle tissue (3, 4). In particular, the type 1 interferon (IFN1) pathway is relevant to DM pathogenesis (5). Overexpression of IFN1-inducible genes has been detected in the muscle, peripheral blood, and skin of patients with DM (5–8). PM is generally considered a prototypic T cell–mediated autoimmune myopathy (9).

Dysferlinopathy, caused by a dysferlin gene mutation, usually presents in late adolescence with muscle weakness; the degenerative muscle changes are often accompanied by inflammatory infiltrates, resulting in misdiagnosis as PM (10). Dysferlin protein appears to play a role in calcium-dependent vesicle-mediated muscle membrane repair (11). The precise pathogenic mechanism underlying dysferlinopathy remains unclear; it is hypothesized that defective membrane repair in dysferlin-deficient muscle leads to muscular dystrophy associated with remarkable muscle inflammation (12). Previous research has shown that the inflammasome pathway components are upregulated and activated in the muscles of patients and mice with dysferlinopathy (13). Dysferlin-deficient muscles also exhibit ubiquitin–proteasomal pathway activation, complement system factor upregulation, and macrophage infiltration (14–16).

Ribonucleic acid sequencing (RNA-Seq) helps detect and quantify complementary deoxyribonucleic acid (cDNA) via next-generation sequencing. In recent years, bioinformatics analysis of gene expression profiles has played a critical role in studying human disease pathogenesis. Differences in the gene expression levels of disease and healthy groups can help detect cellular changes related to disease status, thereby suggesting pathomechanisms (17).

In this study, we aimed to understand the transcriptomic signature and pathomechanism of DM, PM, and dysferlinopathy. We generated transcriptome profiles of muscles from patients with DM, PM, or dysferlinopathy and analyzed their gene expression profiles by using bioinformatics. Additionally, we compared the transcriptome profiles of dysferlinopathy and PM to help identify potential biomarker genes for differentiating between these two clinically similar myopathies because dysferlinopathy is often misdiagnosed as PM.

## 2 Materials and methods

### 2.1 Participant enrollment

We reviewed the medical records from January 2002 to October 2016 in the myopathy database of Gangnam Severance Hospital. The study included 7, 8, 8, and 5 vastus lateralis muscle biopsy samples from 7 patients with DM, 8 patients with PM, 8 patients with dysferlinopathy, and 5 control participants, respectively. The 15 patients with DM or PM had been diagnosed on the basis of the 2017 European League Against Rheumatism/American College of Rheumatology classification criteria for adult and juvenile IIMs (18), by combining the data on clinical characteristics and serological, electrophysiological, and histological evaluations of skeletal muscle biopsies. The diagnosis of dysferlinopathy in the 8 patients had been genetically confirmed. We selected 5 control participants who fulfilled the following criteria: (i) normal muscle pathological features, (ii) normal serum creatine kinase (CK) level, (iii) absence of definite muscle weakness, and (iv) availability of vastus lateralis muscle biopsy sample.

### 2.2 Clinical data collection

Clinical records of all the participants were reviewed to obtain the following information: age at diagnosis, age at onset of symptoms, pattern of weakness on clinical examination, serum CK levels, electrophysiological results, and findings from muscle pathology analysis.

### 2.3 Ribonucleic acid sequencing

cDNA libraries were constructed using the TruSeq RNA library kit, with 1 μg total RNA. The protocol consisted of the following steps: polyA-selected RNA extraction, RNA fragmentation, random hexamer–primed reverse transcription, and 100-nt paired-end sequencing performed using the Illumina HiSeq2500 system. The libraries were quantified using quantitative polymerase chain reaction (qPCR) performed according to the qPCR Quantification Protocol Guide and qualified using an Agilent Technologies 2,100 Bioanalyzer. The sequencer reads were processed and then aligned to the hg19 reference genome by using the STAR v2.7.10a algorithm to perform the alignment (19). STAR is an algorithm for aligning high-throughput short RNA-Seq data to a reference genome; it was developed to overcome the speed and accuracy issue. The reference genome and annotation data were downloaded from the UCSC Table Browser.^1^

For gene annotation, the gencode version 19 annotation file was downloaded from GENCODE.^2^ While running the STAR alignment, “--quantMode” was enabled to create a gene count file. Appropriate read counts were selected on the basis of the sequencing protocol. These raw reads were deposited in the NCBI Sequence Read Archive (SRA) database (accession number: SRP149027).

### 2.4 Bioinformatics analysis

Adaptors were removed from the raw reads by using trim-galore (0.6.6). After the adaptors were trimmed, alignment was performed using an inhouse STAR alignment pipeline by following the recommended procedures (19). The GENCODE version 19 annotation database was used to annotate genes. Read counts, which were obtained from the STAR alignment results, were normalized to compare expression levels between samples by using the DESeq2 package. In addition, DESeq2 identified differentially expressed genes (DEGs). The criteria for identifying DEGs were false discovery rate (FDR) < 0.05 and log2 fold change ≥2 or ≤ −2. Principal component analysis (PCA) was performed to confirm how gene expression in each sample was characterized by pathogenic features. The overall expression pattern in each comparison group was observed using a gene expression heat map.

Gene enrichment analysis of DEGs was performed using Metascape (20).^3^ For coexpression analysis, the protein–protein interaction (PPI) network was constructed using the Metascape website; the results were further visualized using the Cytoscape software (version 3.9.1) (21). Next, molecular complex detection tool (MCODE; version 1.5.1) (22) was utilized to identify the most important modules in the PPI network. The cutoff values were set as follows: node score cutoff = 0.2, maximal depth = 100, K-Core = 2, and degree cutoff = 2. Significant genes were detected using MCODE analysis.

### 2.5 Ethics statement

The institutional review board of Gangnam Severance Hospital, South Korea, approved the research protocol (IRB No. 3–2021-0450). All participants provided written informed consent for clinical data collection and genetic analysis. Access to information that could identify individual participants was prevented during or after data collection.

## 3 Results

### 3.1 Clinical data

Clinical data review for a total of 15 patients with PM or DM confirmed that the diagnosis was acceptable according to the 2017 European League Against Rheumatism/American College of Rheumatology classification criteria (18). The clinical features of the patients with PM or DM have been provided in Table 1.

**Table 1:** Clinical features of patients with polymyositis or dermatomyositis.

Case	Sex	Age (years)	Onset	Clinical feature	CK (U/L)	EMG results	Pathologic findings
PM1	F	35	1MA	Symmetric proximal weakness	3,283	N/A	Myopathic changes
PM2	F	35	2MA	Symmetric proximal weakness	5,670	Myopathic	Endomysial inflammatory cell infiltration
PM3	F	49	1MA	Symmetric proximal weakness	15,216	Myopathic	Endomysial inflammatory cell infiltration
PM4	M	48	7YA	Symmetric proximal weakness	1877	Myopathic	Endomysial inflammatory cell infiltration
PM5	F	21	1YA	Symmetric proximal weakness	2090	Myopathic	Perivascular inflammatory cell infiltration
PM6	F	46	3YA	Symmetric proximal weakness	321	Myopathic	Endomysial inflammatory cell infiltration
PM7	F	48	1MA	Symmetric proximal weakness	13,940	Myopathic	Endomysial inflammatory cell infiltration
PM8	F	25	7MA	Symmetric proximal weakness	12,180	Myopathic	Endomysial inflammatory cell infiltration
DM1	F	38	1YA	Skin lesion† and symmetric proximal weakness	75	Myopathic	Perifascicular atrophy and inflammatory cell infiltration
DM2	F	29	2MA	Skin lesion† and symmetric proximal weakness	584	N/A	Perifascicular atrophy and inflammatory cell infiltration
DM3	F	22	9MA	Skin lesion† and symmetric proximal weakness	2,599	Myopathic	Perifascicular atrophy and inflammatory cell infiltration
DM4	F	27	1YA	Skin lesion† and symmetric proximal weakness	1,178	Myopathic	Perifascicular atrophy and inflammatory cell infiltration
DM5	F	40	4MA	Skin lesion† and symmetric proximal weakness	285	Myopathic	Perifascicular atrophy and inflammatory cell infiltration
DM6	F	42	4MA	Skin lesion† and symmetric proximal weakness	631	Myopathic	Perifascicular atrophy and inflammatory cell infiltration
DM7	M	40	3MA	Skin lesion† and symmetric proximal weakness	13,712	N/A	Inflammatory cell infiltration

PM, polymyositis; DM, dermatomyositis; MA, months ago; YA, years ago; CK, creatine kinase; EMG, electromyography; N/A, not available. ^†^Skin lesions included rash typical of DM (heliotrope rash, Gottron’s papules, Gottron’s sign).

The clinical features of the eight patients with dysferlinopathy were as follows: miyoshi distal myopathy (three patients), limb–girdle muscular dystrophy (three patients), proximodistal type (one patient), and hyperCKemia (one patient). Immunohistochemical analysis of dysferlin showed total loss of expression in seven patients and decreased expression in one patient. Two patients with dysferlinopathy had a history of initial misdiagnosis as PM. The clinical features of the patients with dysferlinopathy have been provided in Table 2.

**Table 2:** Clinical features of patients with dysferlinopathy.

Case	Sex	Age	Onset	Clinical features	Dysferlin expression	Inflammatory cell infiltration	Likely/pathogenic variant
DYS1	F	39	2YA	Miyoshi distal myopathy	Total loss	+	c.663 + 1G > C/c.4886 + 1249G > T
DYS2	F	40	14YA	Miyoshi distal myopathy	Total loss	−	c.2974 T > C/c.2997G > T
DYS3	F	37	3YA	Limb–girdle muscular dystrophy	Total loss	+	c.3032-1G > A/c.3032-1G > A
DYS4	M	38	21YA	Proximodistal type	Total loss	−	c.1284 + 2 T > C/c.2494C > T
DYS5	F	26	5YA	Limb–girdle muscular dystrophy	Total loss	+	c.663 + 1G > C/c.937 + 1G > A
DYS6	F	31	1YA	HyperCKemia	Total loss	−	c.3407delG/c.2633_2634delTT
DYS7	F	36	5YA	Limb–girdle muscular dystrophy	Total loss	+	c.1363_1364delAT/c.757C > T
DYS8	M	40	10YA	Miyoshi distal myopathy	Decreased expression	−	c.1284 + 2 T > C/c.2997G > T

DYS, dysferlinopathy; MA, months ago; YA, years ago.

The control group consisted of two men and three women, with ages ranging from 28 to 47 years. Two patients had been diagnosed with cramps, and three patients had been diagnosed with psychogenic weakness (one patient), psychogenic dystonia (one patient), and myalgia (one patient).

### 3.2 Identification of DEGs

**Figure 1:**
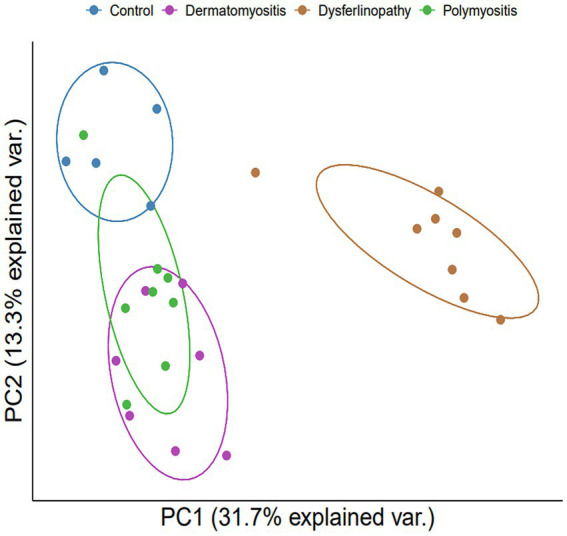
PCA plot between each group of patients and the control. PCA visualized DM and dysferlinopathy as groups distinct from the control. The distinction between PM and the control was ambiguous. var., variance.

**Figure 2:**
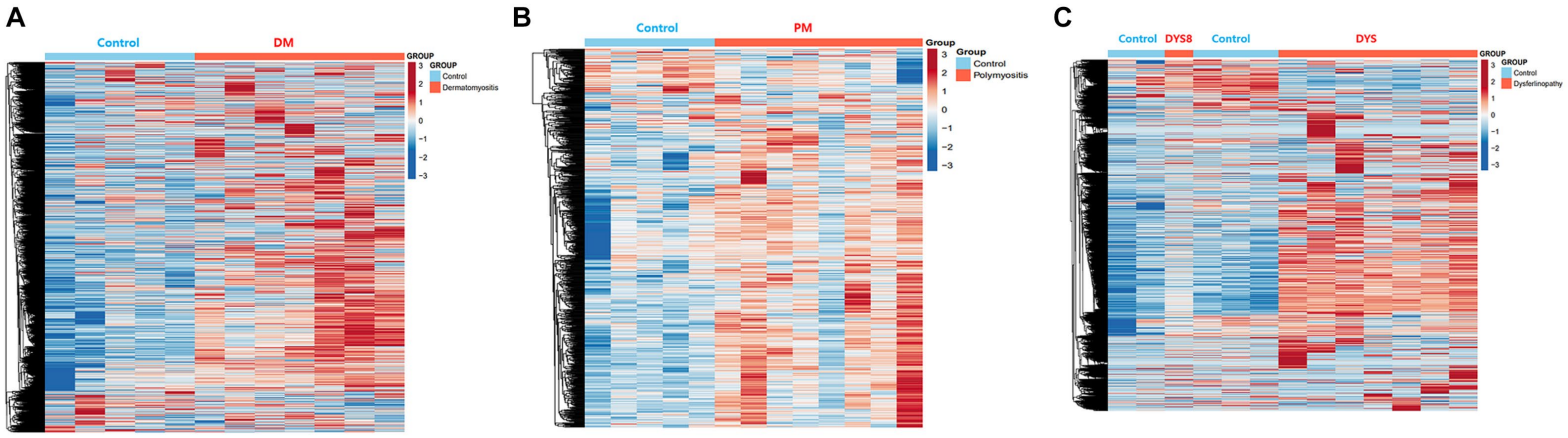
Heat maps of the DEGs of each patient group. **(A)** Heat map of the genes differentially expressed between DM and the control. **(B)** Heat map of the genes differentially expressed between PM and the control. **(C)** Heat map of the genes differentially expressed between dysferlinopathy and the control.

Bioinformatics analysis showed the presence of DEGs in all patient groups. PCA showed differences between the patient and control samples (Figure 1). Patients with DM could be clearly distinguished from those in the control group. Patients with dysferlinopathy were also observed as a distinct group, except for 1 patient (DYS8). In contrast, patients with PM could not be obviously distinguished from controls. The number of DEGs obtained in the analysis was 1,048, 179, and 3,807 for DM, PM, and dysferlinopathy, respectively (Figure 2).

### 3.3 Enrichment analysis of DEGs

**Figure 3:**
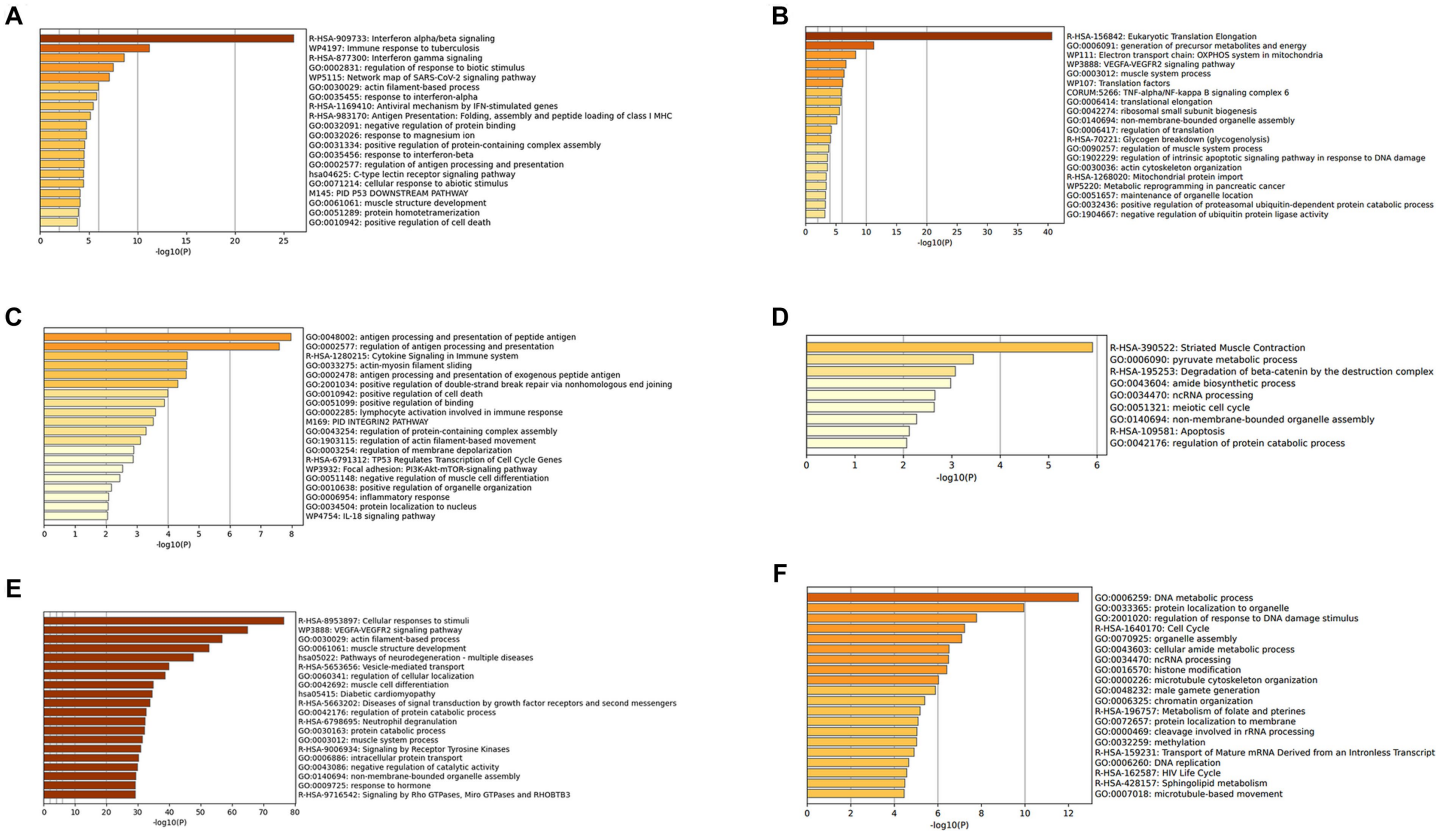
**(A)** Gene enrichment analysis of DEGs upregulated in DM. **(B)** Gene enrichment analysis of DEGs downregulated in DM. **(C)** Gene enrichment analysis of DEGs upregulated in PM. **(D)** Gene enrichment analysis of DEGs downregulated in PM. **(E)** Gene enrichment analysis of DEGs upregulated in dysferlinopathy. **(F)** Gene enrichment analysis of DEGs downregulated in dysferlinopathy.

The DEGs were categorized by gene enrichment analysis, and the top 10 biological pathways of upregulated and downregulated DEGs in patients with DM, PM, or dysferlinopathy have been provided in Figure 3. The results revealed that 576 DEGs were upregulated in DM and were most abundant in the interferon alpha/beta signaling pathway (R-HSA-909733). Twenty-six DEGs mapped to that reactome pathway: *IFI6*, *IFI27*, *IFI35*, *IFIT1*, *IFIT2*, *IFIT3*, *IFIT5*, *IFITM1*, *IFITM3*, *IRF1*, *MX1*, *MX2*, *OAS1*, *OAS2*, *OAS3*, *HLA-A*, *HLA-B*, *HLA-C*, *STAT2*, *ISG15*, *PSMB8*, *SOCS3*, *USP18*, *SAMHD1*, *XAF1*, and *RSAD2.* Additional information regarding these genes is provided in Table 3. The findings showed that 472 DEGs were downregulated in DM; these genes were most abundant in the eukaryotic translation elongation pathway (R-HSA-156842). In addition, 110 DEGs mapped to that reactome pathway.

**Table 3:** Upregulated DEGs involved in the interferon alpha/beta signaling pathway (R-HSA-909733) in DM.

Gene symbol	Function reported to be associated with interferon alpha/beta signaling pathway
*MX1*	IFN1-stimulated gene
*MX2*	IFN1-stimulated gene
*OAS1*	IFN1-stimulated gene
*OAS2*	IFN1-stimulated gene
*OAS3*	IFN1-stimulated gene
*STAT2*	Transcription factor of IFN1-stimulated gene
*ISG15*	IFN1-stimulated gene
*IFI6*	IFN1-stimulated gene
*IFI27*	IFN1-stimulated gene
*IFI35*	IFN1-stimulated gene
*IFIT1*	IFN1-stimulated gene
*IFIT2*	IFN1-stimulated gene
*IFIT3*	IFN1-stimulated gene
*IFIT5*	IFN1-stimulated gene
*IFITM1*	IFN1-stimulated gene
*IFITM3*	IFN1-stimulated gene
*IRF1*	Transcription factor of IFN1-stimulated gene
*HLA-A*	MHC-I formation
*HLA-B*	MHC-I formation
*HLA-C*	MHC-I formation
*PSMB8*	Catalytic subunit of immunoproteasomes, which mediates proteolysis and generates MHC-I molecules
*SOCS3*	Negative IFN1 regulator
*USP18*	Negative IFN1 regulator
*SAMHD1*	IFN1-stimulated gene
*XAF1*	IFN1-stimulated gene
*RSAD2*	IFN1-stimulated gene

IFN1, type 1 interferon; MHC-I, major histocompatibility complex class I.

In the case of PM, 112 DEGs were upregulated and were most abundant in the antigen processing and presentation of peptide antigen pathway (GO:0048002); 24 DEGs mapped to that GO pathway: *B2M*, *CD74*, *PDIA3*, *HLA-C*, *HLA-DRA*, *TAP1*, *TAPBPL*, *CDKN1A*, *GADD45A*, *ITGAL*, *PSMD1*, *ICAM2*, *KIF22*, *SPTBN2*, *TNFRSF14*, *SOCS3*, *DTX3L*, *RHOC*, *ZNF627*, *CREB3L2*, *IFIT3*, *PLAU*, *GSDMD*, and *TBC1D10C*. Additional information regarding these genes has been provided in Table 4. Sixty-seven DEGs were downregulated in PM; these genes were most abundant in the striated muscle contraction pathway (R-HSA-390522). Six DEGs (*MYBPC2*, *TNNC2*, *TNNI2*, *TNNT3*, *SCN9A*, and *MYH1*) mapped to that reactome pathway.

**Table 4:** Upregulated DEGs involved in the antigen processing and presentation of peptide antigen pathway (GO:0048002) in PM.

Gene symbol	Reported function
*B2M*	Involved in MHC-I peptide-loading complex
*CD74*	Essential for assembly and stabilization during HLA class II antigen presentation
*PDIA3*	Involved in MHC-I peptide-loading complex
*HLA-C*	MHC-I formation
*HLA-DRA*	MHC-II formation
*TAP1*	Involved in MHC-I peptide-loading complex
*TAPBPL*	Binds to MHC-I coupled with beta2-microglobulin
*CDKN1A*	Regulator of cell cycle progression
*GADD45A*	Growth arrest and DNA damage–inducible protein
*ITGAL*	Involved in cellular adhesion and costimulatory signaling
*PSMD1*	Degradation machinery of intracellular proteolysis
*ICAM2*	Member of the intercellular adhesion molecule family
*KIF22*	Involved in spindle formation and chromosome movement during mitosis and meiosis
*SPTBN2*	Probably plays an important role in the neuronal membrane skeleton
*TNFRSF14*	Participates in bidirectional cell–cell contact signaling between antigen-presenting cells and lymphocytes
*SOCS3*	Mediates the ubiquitination and subsequent proteasomal degradation of target proteins
*DTX3L*	Plays a role in DNA damage repair and IFN-mediated antiviral responses
*RHOC*	Regulates a signal transduction pathway linking plasma membrane receptors to the assembly of focal adhesions and actin stress fibers
*ZNF627*	May be involved in transcriptional regulation
*CREB3L2*	Transcription factor involved in unfolded protein response
*IFIT3*	IFN-induced antiviral protein
*PLAU*	Specifically cleaves the zymogen plasminogen to form the active enzyme plasmin
*GSDMD*	Precursor of a pore-forming protein that plays a key role in host defense against pathogen infection and danger signals
*TBC1D10C*	Inhibits the Ras signaling pathway via its intrinsic Ras GTPase–activating protein activity

MHC-I, major histocompatibility complex class I; MHC-II, major histocompatibility complex class II; HLA, human leukocyte antigen; IFN, interferon.

In the case of dysferlinopathy, 1,650 DEGs were upregulated; these genes were most abundant in the cellular response to stimuli (R-HSA-8953897). Furthermore, 261 DEGs were mapped to that reactome pathway. The other top-ranked biological pathways were as follows: VEGFA-VEGFR2 signaling pathway (WP3888), actin filament–based process (GO:0030029), muscle structure development (GO:0061061), pathway of neurodegeneration – multiple diseases (hsa05022), and vesicle-mediated transport (R-HSA-5653656). Furthermore, 2,157 DEGs were downregulated in dysferlinopathy; these genes were most abundant in the DNA metabolic process pathway (GO:0006259). Sixty-two DEGs were mapped to that GO pathway. The other top-ranked biological pathways were as follows: protein localization to organelle (GO:0033365), regulation of response to DNA damage stimulus (GO:2001020), cell cycle (R-HSA-1640170), organelle assembly (GO:0070925), and cellular amide metabolic process (GO:0043603).

### 3.4 Identification of key modules and genes, using co-expression analysis

**Figure 4:**
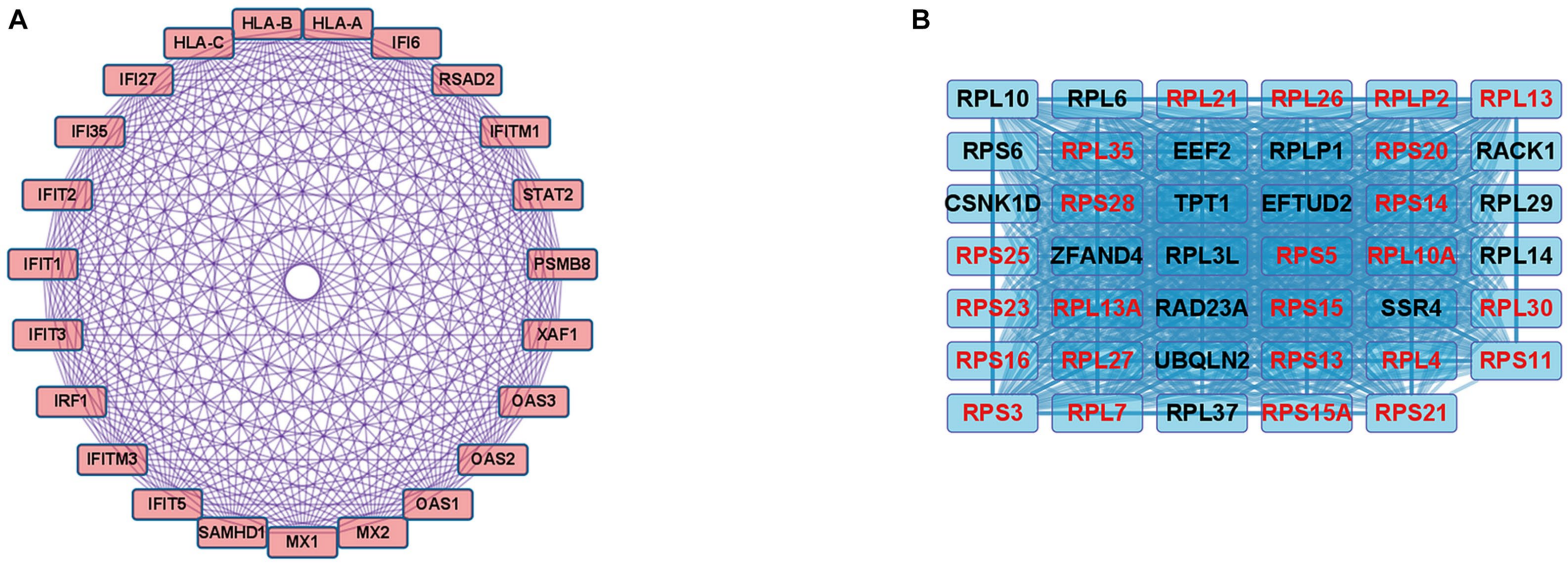
Co-expression analysis of DEGs in DM. **(A)** MCODE analysis of upregulated DEGs reveals the key module containing 23 genes related to the IFN1 signaling pathway (R-HSA-909733). **(B)** MCODE analysis of downregulated DEGs reveals the key module containing 41 genes related to the eukaryotic translation elongation pathway (R-HSA-156842). The 24 genes with the highest degree score of 40 are marked in red.

In the case of the DEGs upregulated in DM, the PPI network comprised 370 DEGs and 1,311 interaction pairs. The MCODE analysis revealed 6 modules, including 71 nodes and 311 pairs. The key module (MCODE score = 11) contained 23 genes related to the interferon alpha/beta signaling pathway (R-HSA-909733), with 253 pairs (Figure 4A). The 23 genes in this module were closely linked to each other, and the degree score of all these genes was 22. These genes corresponded to DEGs mapped to the IFN1 signaling pathway in the gene enrichment analysis.

In the case of the DEGs downregulated in DM, the PPI network comprised 317 DEGs and 2,123 interaction pairs. The MCODE analysis revealed seven modules, including 78 nodes and 874 pairs. The key module (MCODE score = 19) contained 41 genes related to the eukaryotic translation elongation pathway (R-HSA-156842), with 794 pairs (Figure 4B). Of the 41 genes, the following 24 genes had the highest degree score of 40: *RPL10A*, *RPL4*, *RPL7*, *RPL13*, *RPL21*, *RPL26*, *RPL27*, *RPL30*, *RPLP2*, *RPS3*, *RPS5*, *RPS11*, *RPS13*, *RPS14*, *RPS15*, *RPS15A*, *RPS16*, *RPS20*, *RPS21*, *RPS23*, *RPS25*, *RPS28*, *RPL35*, and *RPL13A.*

In the case of DEGs upregulated in PM, the PPI network consisted of 59 DEGs and 63 interaction pairs. The MCODE analysis revealed 2 modules, including 7 nodes and 9 pairs. The key module (MCODE score = 1.5) contained 4 genes (*PDIA3, HLA-C, B2M*, and *TAP1*) related to the antigen processing and presentation pathway (GO:0048002; Figure 5A).

**Figure 5:**
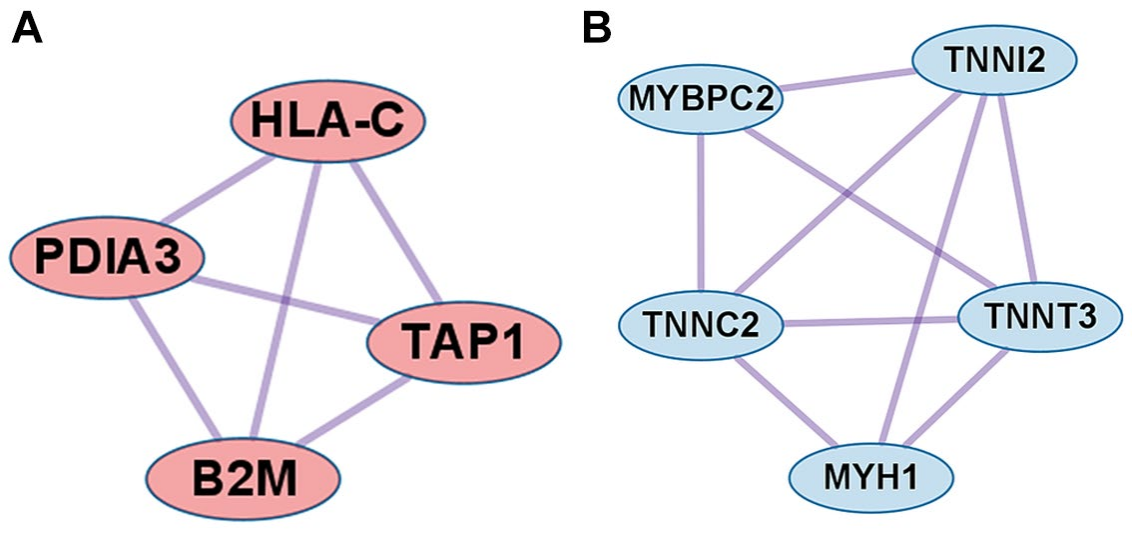
Co-expression analysis of DEGs in PM. **(A)** MCODE analysis of upregulated DEGs reveals the key module containing 4 genes related to the antigen processing and presentation of peptide antigen pathway (GO:0048002). **(B)** MCODE analysis of downregulated DEGs reveals the key module containing 5 genes related to the striated muscle contraction pathway (R-HSA-390522).

In the case of DEGs downregulated in PM, the PPI network consisted of 27 DEGs and 32 interaction pairs. The MCODE analysis results revealed 2 modules, including 8 nodes and 12 pairs. The key module (MCODE score = 1.7) contained 5 genes (*MYBPC2*, *TNNI2*, *TNNT3*, *TNNC2*, and *MYH1*) related to the striated muscle contraction pathway (R-HSA-390522; Figure 5B).

In the case of dysferlinopathy, no network was identified in the PPI analysis of up- or downregulated DEGs.

### 3.5 Identification of hub genes within important biological pathways in dysferlinopathy

**Figure 6:**
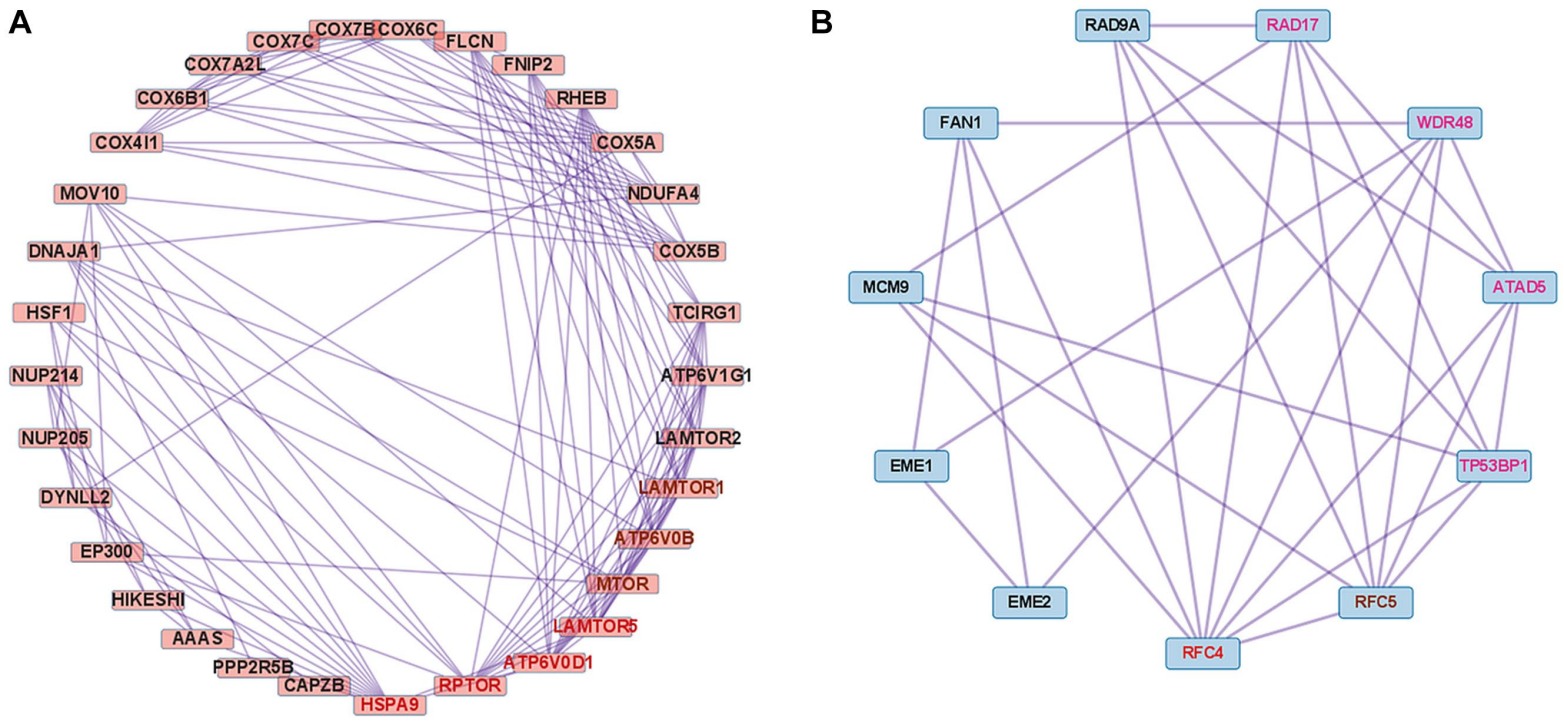
Co-expression analysis of DEGs mapped to important pathways in dysferlinopathy. **(A)** PPI with 261 upregulated DEGs related to the cellular response to stimuli (R-HSA-8953897). MCODE analysis reveals the key module containing 32 genes related to the cellular response to stress (R-HSA-2262752). The 4 genes with the highest degree score of 13 are marked in red, and the 3 genes with a score of 12 are marked in brown. **(B)** PPI with 62 downregulated DEGs related to the DNA metabolic process (GO:0006259). MCODE analysis reveals the key module containing 11 genes related to the cellular response to DNA damage stimulus (GO:0006974). The gene with the highest degree score of 8 is marked in red, the gene with a score of 7 in brown, and the 4 genes with a score of 6 in pink.

We performed PPI analysis with 261 upregulated DEGs related to the cellular response to stimuli (R-HSA-8953897) to identify hub genes in this pathway. This PPI network comprised 261 DEGs and 5,420 interaction pairs. The MCODE analysis revealed 8 modules, including 185 nodes and 2,891 pairs. The key module (MCODE score = 4.12) contained 32 genes related to the cellular response to stress (R-HSA-2262752), with 134 pairs. Of the 32 genes, 4 (*HSPA9, RPTOR, ATP6V0D1*, and *LAMTOR5*) had the highest degree score of 13 and 3 (*MTOR, LAMTOR1*, and *ATP6V0B*) had a score of 12 (Figure 6A).

We performed PPI analysis with 62 downregulated DEGs related to the DNA metabolic process (GO:0006259) to identify hub genes in this pathway. The PPI network comprised 43 DEGs and 117 interaction pairs. The MCODE analysis revealed 3 modules, including 20 nodes and 40 pairs. The key module (MCODE score = 2.63) contained 11 genes (*ATAD5*, *RAD9A*, *RAD17*, *TP53BP1*, *RFC5*, *RFC4*, *MCM9*, *WDR48*, *FAN1*, *EME1*, and *EME2*) related to the cellular response to DNA damage stimulus (GO:0006974), with 29 pairs. Of the 11 genes, *RFC4* was found to have the highest degree score of 8 (Figure 6B).

### 3.6 Comparative analysis between PM and dysferlinopathy

**Figure 7:**
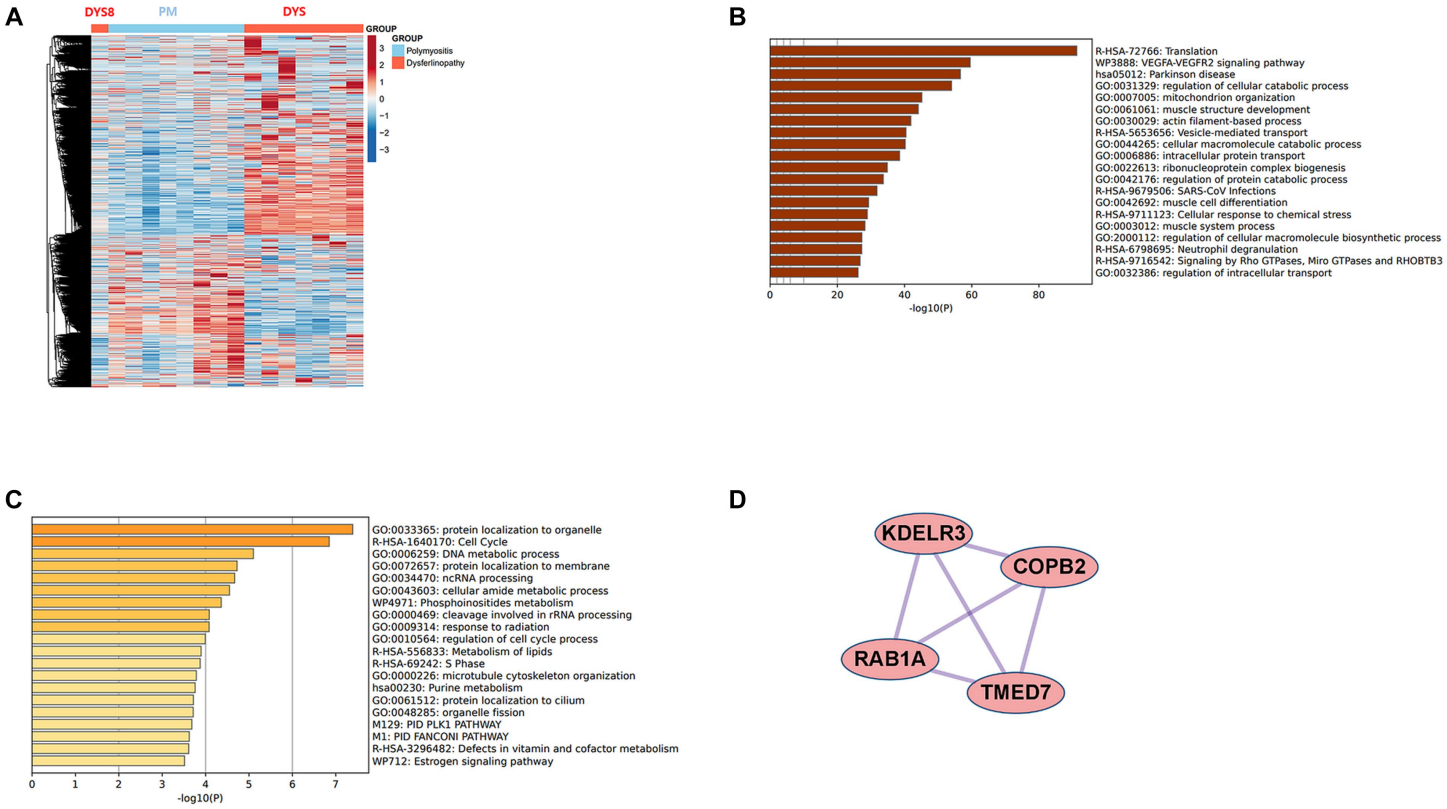
Comparative analysis between PM and dysferlinopathy. **(A)** Heat map of the genes differentially expressed between PM and dysferlinopathy. **(B)** Gene enrichment analysis of DEGs upregulated in dysferlinopathy. **(C)** Gene enrichment analysis of DEGs upregulated in PM. **(D)** Co-expression analysis of DEGs mapped to the protein localization to organelle pathway (GO:0033365) in PM. MCODE analysis shows 1 module, including 4 genes related to endoplasmic reticulum to Golgi vesicle–mediated transport (GO:0006888).

The results revealed that 2,869 DEGs were upregulated in dysferlinopathy and 1,444 DEGs in PM (Figure 7A). PPI analysis of the upregulated DEGs did not identify any networks for dysferlinopathy or PM. Gene enrichment analysis results for the DEGs upregulated in dysferlinopathy and PM have been presented in Figures 7B,C, respectively. The DEGs upregulated in dysferlinopathy were most abundant in the translation pathway (R-HSA-72766), and 775 DEGs were mapped to this reactome pathway. PPI analysis of the 775 DEGs mapped to the translation pathway (R-HSA-72766) did not identify any network. The DEGs upregulated in PM were most abundant in the protein localization to organelle pathway (GO:0033365), and 58 DEGs were mapped to this GO pathway. PPI analysis of 58 DEGs mapped to the protein localization to organelle pathway (GO:0033365) showed that the network was composed of 35 DEGs and 42 pairs. The MCODE analysis results revealed 1 module, including 4 nodes and 6 pairs (Figure 7D). These 4 genes (*KDELR3*, *C0PB2*, *TMED7*, and *RAB1A*) were related to endoplasmic reticulum (ER) to Golgi vesicle–mediated transport (GO:0006888).

## 4 Discussion

In this study, we performed bioinformatics analysis of the gene expression profiles obtained for the muscles of patients with DM, PM, or dysferlinopathy. Gene enrichment analysis revealed that upregulated DEGs were most abundant in the IFN1 signaling pathway in DM and the antigen processing and presentation of peptide antigen pathway in PM. The DEGs upregulated in dysferlinopathy were most abundant in the cellular response to stimuli.

### 4.1 IFN1 signaling pathway in DM

**Figure 8:**
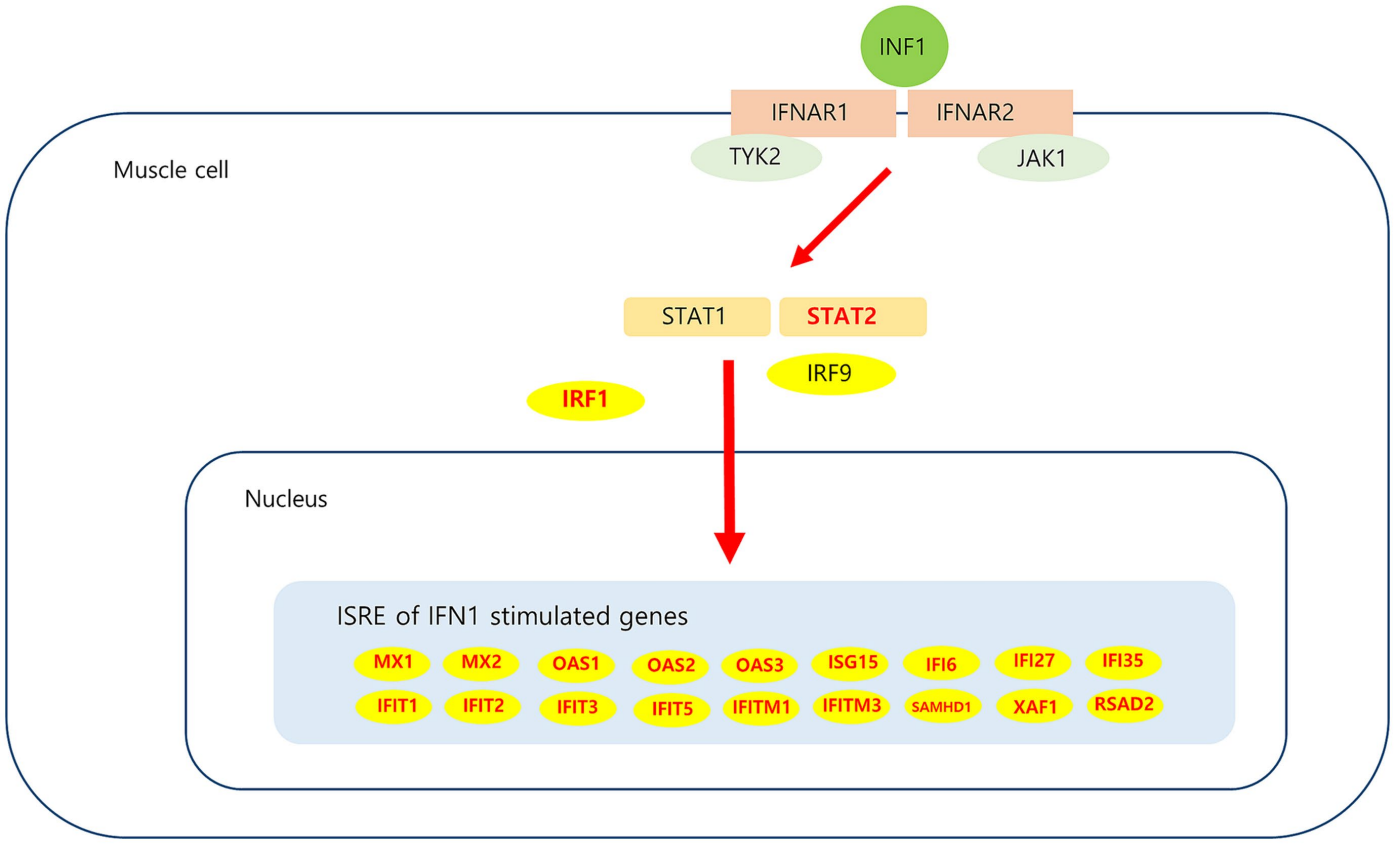
Upregulated DEGs associated IFN1 signaling pathway in DM. IFN1 signal via the IFNAR (interferon-alpha/beta receptor)-1 and IFNAR-2, which are expressed in muscle cell activate JAK/STAT pathway. Subsequent JAK1 and TYK2-dependent phosphorylation of IFNAR creates docking sites for STAT1 and STAT2. Phosphorylation of STAT1 and STAT2, which leads to heterodimerization and together with IRF9 to the formation of ISGF3. This heterotrimeric complex translocates to the nucleus and binds the ISRE (interferon-sensitive response element) sequence present in ISGs (interferon stimulated genes). Upregulated DEGs in this study is marked in red.

Analysis of muscle transcripts from patients with DM helped identify 26 upregulated DEGs involved in the interferon alpha/beta signaling pathway. Of these, 18 were IFN1-stimulated genes (ISGs), which included 2 ISG transcription factor–encoding genes (*STAT2* and *IRF1*), 2 IFN1 negative regulator–encoding genes (*SOCS3* and *USP18*), and 3 genes involved in MHC-I formation (*HLA-A*, *HLA-B*, and *HLA-C*). These upregulated DEGs have been schematized in Figure 8.

IFN1-related proteins are prominent in DM muscles and are considered a potential DM diagnostic biomarker (23, 24). Additionally, the IFN1 pathway biomarker in blood is highly correlated with clinical severity in DM (25). Analysis of different subgroups for DM muscle biopsies has shown that the IFN1 signature is a useful unifying pattern for all DM subgroups and can be used to differentiate DM from other diseases (26).

The ISGs such as *MX*, *ISG15*, *OAS*, *IFITM*, *IFIT*, and *IFI* observed in our results have also been reported as hub genes in a bioinformatics study performed using muscle from a patient with DM (27–29). Myxovirus resistance protein 1 (MxA) is a key mediator of the interferon-induced antiviral response against a variety of viruses. Sarcoplasmic MxA expression has been shown to be a DM hallmark, and the European NeuroMuscular Center (ENMC) 2018 DM classification criteria include perifascicular MxA overexpression as definite DM muscle biopsy findings (26). *ISG15* is one of the most strongly induced ISGs; A previous study proposed that the *ISG15* expression level in muscle alone could be used to reliably quantify IFN1 pathway activation in adult inflammatory myositis (30). In patients with juvenile DM, muscle *ISG15* expression was found to be inversely correlated with the clinical and histological severity of the disease; these results suggest that *ISG15* negatively regulates the IFN1 signaling pathway (31). Thus, *ISG15* may be used as a biomarker for DM diagnosis and monitoring; further research on the use of *ISG15* for this purpose is expected. The expression of *USP18* and *SOCS3*, which are negative regulators of IFN1 pathway signaling (31–33), was also found to increase in the current study. The possibility of their involvement in regulating sustained IFN1 signaling pathway activation in DM can be considered. Among the other genes analyzed in this study, *STAT2* and *IRF1* which are ISG transcription factors, have been reported to be overexpressed in DM (34).

The mechanisms underlying IFN1 induction in DM remain unclear. IFN1 is secreted from dendritic cells because of Toll-like receptor (TLR) induction and from muscle cells because of retinoic acid–inducible gene 1 (RIG-1) activation (35). Direct response by a viral pathogen or secondary response associated with muscle tissue remodeling may cause TLR induction (36). An *in vitro* study showed that hypoxia triggers IFN1 production in muscles (37).

The IFN1 pathway plays an important role with respect to muscle fibers and vascular injury in DM pathophysiology (5, 38, 39). Autocrine IFN1 signaling was recently found to prevent muscle stem cell proliferation in DM, leading to muscle repair deficit (40). These *in vitro* pathogenic effects of IFN1 in muscle and endothelial cells can be prevented by pharmacologic blockade of IFN1 signaling by using JAK inhibitors or anti-IFN receptor antibodies (39, 40). A systematic literature review suggested that JAK inhibitors represent a viable treatment option for DM (41).

### 4.2 Antigen processing and presentation of peptide antigen pathway in PM

Analysis of muscle transcripts from patients with PM helped identify 4 upregulated DEGs (*PDIA3, TAP1, B2M*, and *HLA-C*) involved in antigen processing and peptide antigen presentation. All these genes encode proteins involved in MHC-I formation and quality control. Normal muscle fibers do not express MHC-I, but MHC-I overexpression has been histopathologically confirmed in the muscles of patients with PM or IBM, and the inflammatory response by the MHC-I–CD8 complex is a major pathological mechanism in PM (42, 43). Thus, our findings are consistent with those of previous studies.

**Figure 9:**
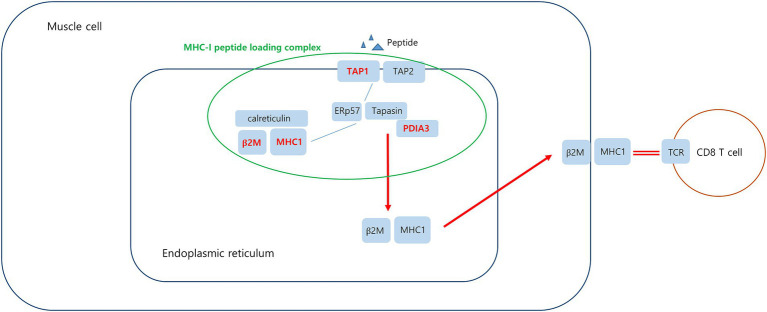
Upregulated DEGs associated MHC-I peptide loading complex (PLC) in PM. Peptide-receptive MHC-I heterodimers are recruited by calreticulin to the PLC. The peptide transporter TAP1/2 shuttles peptides from the cytosol into a molecular basket formed by the editing modules. After tapasin-facilitated peptide loading and proofreading, MHC-I are released from the PLC and traffic via the Golgi compartment to the cell surface. Upregulated DEGs in this study is marked in red.

Protein disulfide-isomerase A3 (*PDIA3*), transporter associated with antigen processing 1 (*TAP1*), and beta2-microglobulin (*B2M*) are involved in the MHC-I peptide loading complex (PLC) (44, 45). The PLC, a transient multisubunit membrane complex in the ER, is essential for establishing stable peptide–MHC-I complexes by stringent peptide proofreading and quality control processes (45). TAP, a central part of the PLC, ensures high local concentrations of translocated peptides in the ER lumen proximal to the PLC (45, 46). PDIA3 forms a disulfide-linked complex with tapasin as a component of the MHC-I loading complex, which is thought to either stabilize the complex or facilitate correct class I molecule assembly (47). These upregulated DEGs have been schematized in Figure 9.

### 4.3 Cellular response to stress in dysferlinopathy

Analysis of muscle transcripts from patients with dysferlinopathy helped identify the following 7 upregulated DEGs involved in the cellular response to stress: *HSPA9*, *RPTOR*, *MTOR*, *LAMTOR1*, *LAMTOR5*, *ATP6V0D1*, and *ATP6V0B*.

#### 4.3.1 HSPA9

*HSPA9* encodes mortalin, a 74 kDa mitochondrial-resident protein also known as p66mot-1, mitochondrial stress-70 protein (mtHsp70), peptide-binding protein 74 (PBP74), and glucose-regulated protein 75 (GRP75) (48, 49). Mortalin is located in the mitochondria, ER, plasma membrane, cytoplasmic vesicles, and cytosol (50). Its activity and function are determined by its cellular localization and binding partners (48).

**Figure 10:**
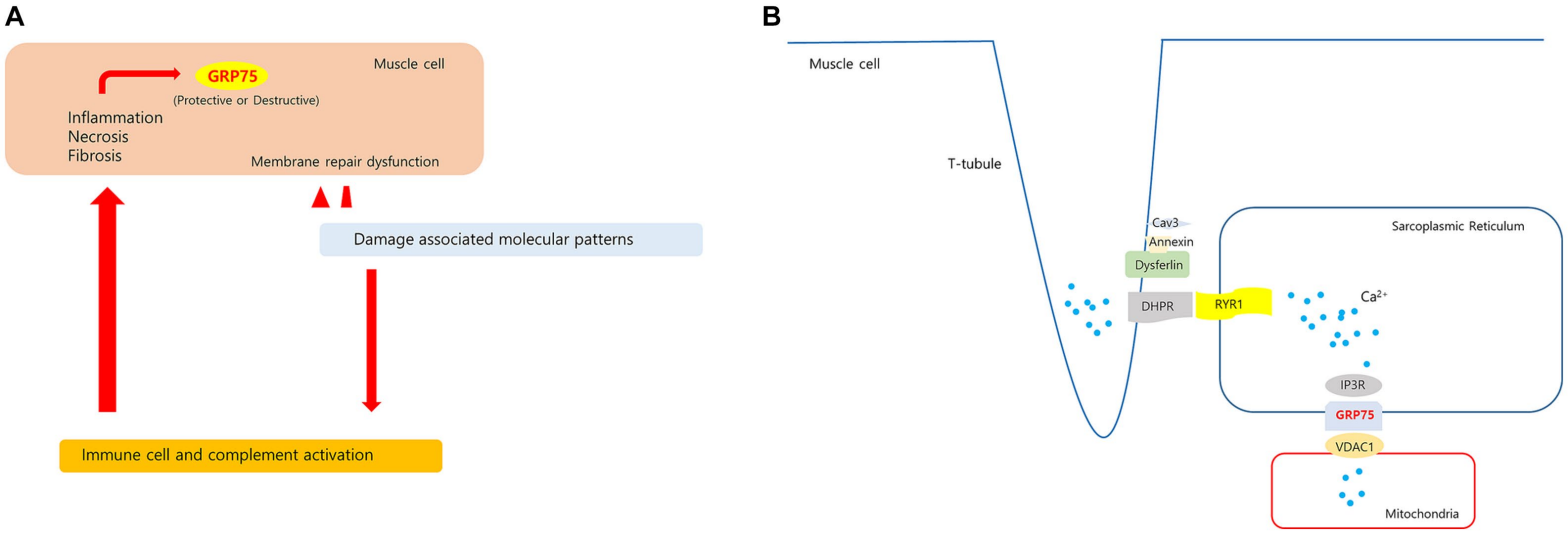
Hypotheses regarding upregulated *HSPA9* in dysferlinopathy. **(A)** Membrane damage to muscle fibers with membrane repair dysfunction causes a prolonged releases of damage associated molecular patterns. These molecules stimulate leukocytes to produce pro-inflammatory cytokines and activate the complement system. The sustained inflammatory signaling may cause increased GRP75 in muscle cell. **(B)** In proposed model of dysferlin as a Ca^2+^-sensitive signaling scaffold localized to the t-tubule membrane, that is designed to respond to changes in intracellular Ca^2+^ caused by t-tubule membrane stress and damage. The SR(sarcoplasmic reticulum)-mitochondria Ca^2+^ transport is mediated by the macromolecular complex IP3R-GRP75-VDAC1. *HSPA9* overexpression may be associated with changes in Ca^2+^ homeostasis in dysferlinopathy.

GRP75 (mortalin) levels in human skeletal muscles increase with oxidative stress (51, 52). GRP75 expression has been found to increase in the muscles of patients with inflammatory myositis. GRP75 levels were found to increase in nonregenerating myofibers of patients who had myositis and were positive for sarcolemmal MHC-I immunoreactivity (53). GRP75 is also involved in mediating endo/sarcoplasmic reticulum (ER/SR)–mitochondria Ca^2+^ transport by forming the inositol 1,4,5-triphosphate receptor (IP3R)–GRP75–voltage-dependent anion channel 1 (VDAC1) complex in skeletal muscle cells (54). Changes in ER/SR-mitochondria Ca^2+^ transport may be related to impaired skeletal muscle function, such as in Duchenne muscular dystrophy (DMD) (55). Dysferlin is involved in T-tubule biogenesis and Ca^2+^ homeostasis maintenance (56–58). *HSPA9* overexpression may be related to changes in intramuscular Ca^2+^ homeostasis. *HSPA9* has varying functions depending on the cell type and interacting proteins; therefore, further research is required to clarify the significance of intramuscular *HSPA9* expression in dysferlinopathy. Hypotheses regarding upregulated *HSPA9* in dysferlinopathy has been schematized in Figure 10.

#### 4.3.2 mTOR1 pathway

**Figure 11:**
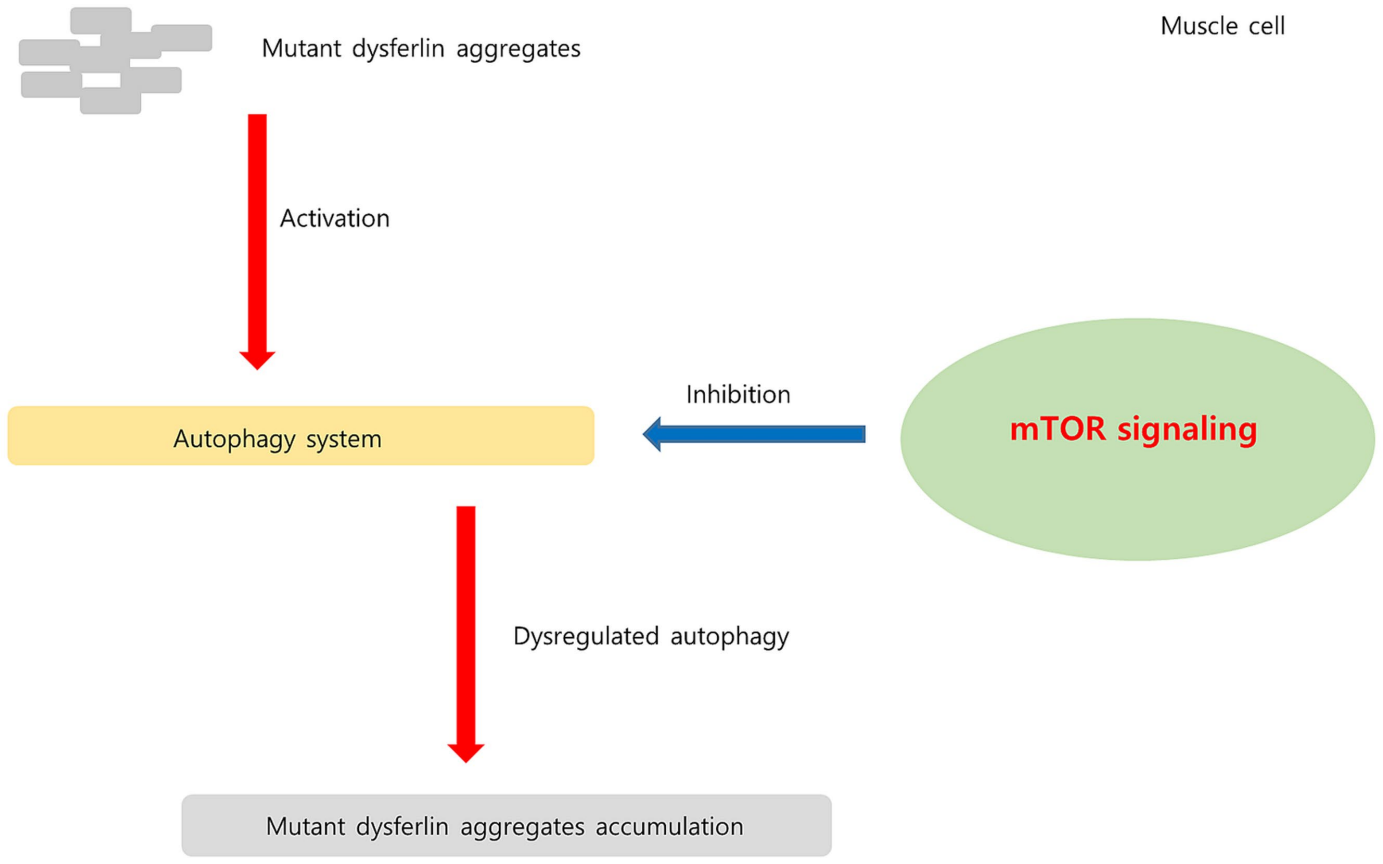
Hypothesis regarding upregulated mTOR signaling pathway in dysferlinopathy. In dysferlinopathy, the autophagy program is activated for the degradation of mutant dysferlin aggregates. Chronic mTOR1 signaling inhibits autophagic activity. Upregulated mTOR pathway may be associated with dysregulated autophagy in dysferlinopathy.

Regulatory-associated protein of MTOR complex 1 (RPTOR; *RPTOR*), *MTOR*, and late endosomal/lysosomal adaptor, MAPK, and MTOR activator (*LAMTOR*) are associated with the mTOR pathway (59). mTORC1 acts as an upstream regulator of autophagy induction in skeletal muscle (60). Chronic mTORC1 activation in old muscle leads to muscle atrophy mainly because of inability to induce autophagy (61). Autophagy dysregulation is related to various hereditary myopathies, including autophagic vacuolar myopathies (60). Autophagy was found to be impaired in patients with DMD and in mdx mouse models. In mdx mice, mTOR was constitutively activated, leading to autophagy-inducing gene downregulation at the molecular level (62). In dysferlinopathy, the autophagy program has been found to be activated for degradation of mutant dysferlin aggregates (15). Inhibition of mutant dysferlin aggregate formation in the ER by rapamycin, an mTOR inhibitor, has been reported in a cellular model (63). A recently published bioinformatics analysis involving dysferlinopathy identified the genes involved in the ubiquitin–proteasome pathway as key genes (64). Thus, our results suggest that the mTORC1 and autophagy pathways play a role in dysferlinopathy pathogenesis. The hypothesis of upregulated mTOR1 signaling in dysferlinopathy has been schematized in Figure 11.

### 4.4 DEGs downregulated in DM, PM, and dysferlinopathy

Twenty-four ribosome-associated genes were found to be downregulated on analyzing muscle transcripts from patients with DM. Decreased ribosomal-associated gene expression has recently been detected in the muscle and skin in juvenile DM (65). Analysis of muscle transcripts from patients with PM helped identify 5 downregulated DEGs (*MYBPC2*, *TNNI2*, *TNNT3*, *TNNC2*, and *MYH1*) related to striated muscle contraction. These findings may reflect muscle degeneration in DM and PM.

Analysis of muscle transcripts from patients with dysferlinopathy identified 6 downregulated DEGs (*RFC4*, *RFC5*, *ATAD5*, *TP53BP1*, *WDR48*, and *RAD17*) related to the cellular response to DNA damage stimulus. Replication factor C (RFC) plays an important role in DNA replication and repair, cell proliferation, cell cycle checkpoint regulation, and cell growth under stress (66). However, the implications of reduced RFC expression in dysferlinopathy require further study because association between myopathy and RFC expression has not been reported.

### 4.5 Comparative analysis between PM and dysferlinopathy

Comparative analysis between PM and dysferlinopathy revealed that the DEGs upregulated in PM were most abundant in the protein localization to organelle pathway and helped identify four genes (*KDELR3*, *C0PB2*, *TMED7*, and *RAB1A*) that were related to ER to Golgi vesicle–mediated transport. However, no promising pathway or gene that could be used as a biomarker for differentiating PM from dysferlinopathy was identified.

### 4.6 Limitations of this study

This study has several limitations. First, myositis-specific antibodies were not tested in patients with DM or PM. Additionally, patients with PM were not obviously distinguished from control, which may be related to the debatable issue about the real existence of PM as a separate pathobiological entity. PM is reported to be an overdiagnosed entity as subgroups of inflammatory myopathy are subdivided. Therefore, confirmation with a complete myositis autoantibody panel is recommended for the diagnosis of PM (67). However, we could not perform autoantibody test because we did not have serum samples from the patients. Second, 1 patient with dysferlinopathy (DYS8) showed a different gene expression pattern among the dysferlinopathy groups. This result was consistent with the pathological findings for dysferlin expression. Dysferlin expression was completely lost in the other patients; however, only this patient (DYS8) showed reduced expression. For the interpretation of this results, it would be helpful to collect additional dysferlinopathy cases in which the dysferlin was expressed and analyze the intramuscular transcriptome profiles of these patients. Third, the sample size of the dataset used in this study was small; therefore, the results obtained from the bioinformatics analysis should be further verified. Fourth, several of the hub genes identified in this study have not been previously reported to be associated with dysferlinopathy; therefore, further *in vitro* and functional studies are required to verify the molecular biological mechanisms of the genes identified in this study.

## 5 Conclusion

We identified differential biological pathways on the basis of bioinformatics analysis of gene expression profiles obtained from the muscles of patients with DM, PM, or dysferlinopathy. Overexpression of genes related to the IFN1 signaling pathway was identified in DM, and overexpression of genes related to MHC-I formation and quality control was identified in PM. In dysferlinopathy, overexpression of *HSPA9* and the mTORC1 signaling pathway genes was detected. Further studies are required to verify the role of *HSPA9* and the mTORC1 pathway in the pathomechanism of dysferlinopathy.

## Data availability statement

The datasets presented in this study can be found in online repositories. The names of the repository/repositories and accession number(s) can be found at: https://www.ncbi.nlm.nih.gov/, SRP149027.

## Ethics statement

The studies involving humans were approved by the institutional review board of Gangnam Severance Hospital, South Korea, approved the research protocol (IRB No. 3–2021-0450). The studies were conducted in accordance with the local legislation and institutional requirements. The participants provided their written informed consent to participate in this study.

## Author contributions

H-NJ: Conceptualization, Data curation, Formal analysis, Investigation, Methodology, Writing – original draft, Writing – review & editing. TL: Conceptualization, Data curation, Formal analysis, Investigation, Methodology, Writing – original draft, Writing – review & editing. HP: Conceptualization, Data curation, Methodology, Supervision, Writing – review & editing. YY: Supervision, Writing – review & editing. S-HO: Supervision, Writing – review & editing. S-WK: Supervision, Writing – review & editing. Y-CC: Conceptualization, Data curation, Formal analysis, Investigation, Methodology, Supervision, Writing – review & editing.

## Footnotes

^1^ http://genome.ucsc.edu

^2^ https://www.gencodegenes.org

^3^ http://metascape.org
